# Environmental Resource Management in Borderlands: Evolution from Competing Interests to Common Aversions

**DOI:** 10.3390/ijerph120707541

**Published:** 2015-07-06

**Authors:** Patrick Henry Buckley, John Belec, Jason Levy

**Affiliations:** 1 Huxley Environmental College, Western Washington University, 516 High St., Bellingham, WA 98225, USA; 2 Department of Geography and the Environment, University of the Fraser Valley, Abbotsford, BC V2S 7M7, Canada; E-Mail: John.Belec@ufv.ca; 3 L. Douglas Wilder School of Government and Public Affairs, Virginia Commonwealth University, 923 W. Franklin St., Box 842028, Richmond, VA 23284, USA; E-Mail: jklevy@vcu.edu

**Keywords:** cross border regions, cross border resource management, game theory, Canada-United States border region

## Abstract

Great enthusiasm is attached to the emergence of cross-border regions (CBRs) as a new institutional arrangement for dealing with local cross-border environmental resource management and other issues that remain too distant from national capitals and/or too expensive to be addressed in the traditional *topocratic* manner requiring instead local *adhocratic* methods. This study briefly discusses the perceived value of CBRs and necessary and sufficient conditions for the successful and sustainable development of such places. Then, assuming that necessary conditions can be met, the study investigates an intriguing hypothesis concerning the catalyzing of sustainable consensus for cross-border resource management based on a game theoretical approach that employs the use of dilemma of common aversion rather than the more traditional dilemma of competing common interests. Using this lens to investigate a series of events on the Pacific northwestern Canadian-American border in a part of the Fraser Lowland, we look for evidence of the emergence of an active and sustainable CBR to address local trans-border resource management issues. Although our micro-level scale fails to conclusively demonstrate such evidence, it does demonstrate the value of using this approach and suggests a number of avenues for further research.

## 1. Introduction

Cross-border security policies can raise competing and contradictory agendas especially when viewed through the lens of environmental resource management and sustainability. For example, increased border infrastructure and redundancy in economic systems that provide trans-border services, like electrical power generation and distribution, can be seen as a means of increasing societal safety and resilience. However, they can also increase stress on local ecological systems negatively impacting on the health and safety of humans and other species residing in a region [1,2] including situations when trans-border power generation are considered [3]. Unfortunately, Betsill [4] has suggested that no multi-lateral institution such as the Commission for Environmental Cooperation (CEC) is currently fully capable of addressing this situation in part due to the fact that solutions will require a multi-level approach [5]. Further, under these circumstances it is common that what is deemed beneficial to one border partner might prove to be injurious to the other [6]. To begin to address this conundrum, this study will use a case study approach and limit its scope to only the local regional level, leaving the national for later investigation. Then, two promising avenues will be investigated and intertwined. These are Cross Border Regions (CBR) as the institutions that further local level Cross Border Cooperation [7], and second the catalyzing of sustainable consensus for cross-border resource management based on a game theoretical approach that employs the use of dilemma of common aversion rather than the more traditional dilemma of competing common interests [8].

## 2. Cross Border Regions

### 2.1. Definition

Great enthusiasm is attached to the emergence of cross-border regions (CBRs) as a new institutional arrangement for dealing with local cross-border issues that remain geographically, financially, or politically too distant from capitals to be addressed in the traditional *topocratic* manner [9]. Instead, the CBR arrangement promises a new adhocratic means that allows local stakeholders to address local issues even in this era of increased security. The major stimuli for this change is the combination of the end of the Cold War and the deterritorialization/reterritorialization tendencies of advanced capitalism, leaving borderlands throughout the world, under increased freedom and great pressure to create more efficient and integrated regions. However, despite all the hype, in a very real sense the success of CBRs faces the same dialectic of cooperation *vs.* conflict that has long hampered nation to nation relationships.

This then leads to a key issue in this paper the (often vague) distinction between cross-border “space” *vs.* cross-border “integration”. In theory, CBRs co-exist with international boundaries. In practice, the term is usually reserved for trans-boundary regions where there is some evidence of integration and/or cooperation, and thereby also a CBR consciousness. Even with these however, there is a difference regarding the degree to which a given CBR is functionally integrated [10].

Keeping all of this in mind and drawing on Scott [11,12], Buckley and Belec [13] have proposed that there are both necessary and sufficient conditions that lead to a successful and sustainable CBR. Of these the necessary conditions can be either endogenously created or exogenously stimulated by central actors, as occurred in the European Union over the past decades. However, meeting the critical sufficient conditions can only be demonstrated ex post facto through sustained activity benefiting the stakeholders on both sides of the border. Thus, it is this nexus between necessary and sufficient conditions which this paper wishes to explore. Specifically are there triggers that can help stimulate local cooperation while ameliorating conflict that results in a CBR that produces positive sustainable change for the majority of its stakeholders?

This paper proposes to explore this question using aspects of game theory that Ali [8] has suggested in his national level study for “catalyzing sustainable consensus” in borderlands for resource management which draws on the work of Stein [14]. He employs the use of dilemma of common aversion rather than the more traditional dilemma of competing common interests as defined in game theory. Although the focus of our study is on a particular instance of cross-border resource management and its relationship to CBR development, one should recognize that Ali [8,15] is concerned with a much broader issue of catalyzing consensus around conflicts nationally or locally. Specifically, he proposes that engagement focused on environmental issues especially when led by planners could foster an “operational arena” within which a multitude of potentially long standing conflicts between places, many of which can threaten not merely destructive environmental actions but even outright violence, could be mediated and addressed. This implies the creation of “superordinate goals”, issues which require the two or more potentially adversarial groups to be jointly involved in order to accomplish a successful outcome. Ali proposed that three separate paths can lead to such a situation ([8], p. 173):

1. “*Framing [environmental] conflicts as a dilemma of common aversion*”
2. “*Linking environmental concerns to other issues*”
3. “*Using environmental concerns as a trust-building tool*”

In the American-Canadian context addressed here at the local regional level, cross-border conflicts fortunately do not have a cloud of violence looming over them. However, a CBR by its very definition is closely related to the concept of building an operational arena that brings together two groups divided by a border and facing superordinate goals. In fact, Blatter [10,16] has extensively detailed how the use of environmental threats to Lake Constance led to the creation of a German, Austrian, and Swiss CBR to address conflicts of local aversion and provided the basis for addressing further issues. The Fraser Lowland has many more issues then just airshed impacts and power plant siting that will need to be addressed over time, again our study seeks to see how far along this region is in meeting sufficient conditions to demonstrate its successful transition into a viable CBR.

**Figure 1:**
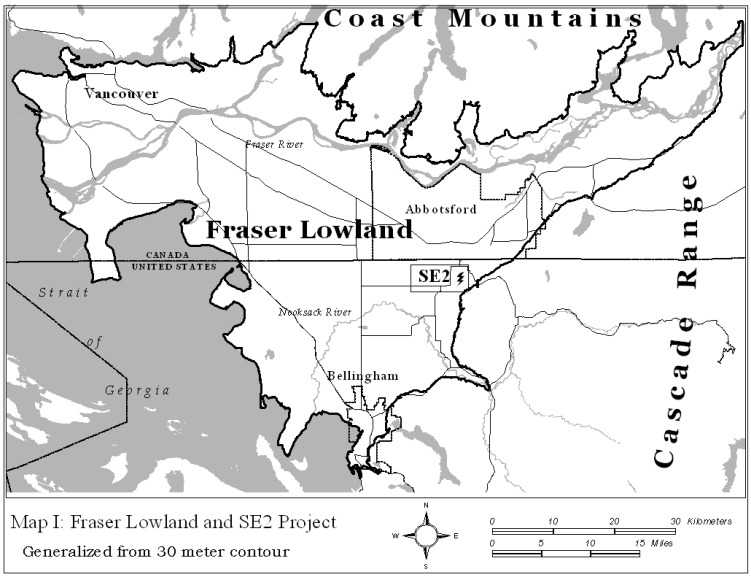
Map of fraser lowland and location of se2 project.

Specifically we will use this lens to investigate a series of events on the Canadian-American border in a part of the Fraser Lowland, a roughly triangular region divided between the two nations and anchored by port cities of Vancouver, BC to the north, Bellingham, WA to the south and the Fraser Valley to the east (see Figure 1).

### 2.2. Necessary and Sufficient Conditions for a CBR

The study of state borders and borderlands has a long history in the discipline of Geography [17]. In the past decade, trends like the expansion of the European Union and globalization have redefined the role of borders. The creation of Eurozones, for example, represents an explicit attempt to foster cross-border identities [10,11,12].

Within this context, CBRs have received increased scholarly interest [7,10,12,18]. As Perkmann and Sum note, although CBRs share the same history as borders, “*(w)hat is new…is that the construction of cross-border regions have become a more or less explicit strategic objective pursued by various social forces within and beyond border regions…*” ([7], p. 3).

One of the most intriguing yet problematic aspects of CBRs is their plentitude of institutional designs and strategies. This complexity is further exhibited both in the great varieties of transnational regionalism that exists and also the “*…myriad of systems operating at various spatial levels…*” ([12], p. 606) within a given region. This results in the emergence of multiple, episodic, and ad hoc forms of cooperative and coordinated governance networks across borders that are both overlapping and interconnected. Leresche and Saez [9] have likened this to the biological concept of synapsis or “*'very fine communication between neighboring cells through small networks in a membrane' or 'a point of contact between two neurons'*” (Dictionary Robert, reported in Leresche and Saez ([9], p. 88)). Over time this micro level cross-border activity can lead to a *broadening* and *thickening* of relationships and the eventual emergence of CBR governance.

Despite the multitude of CBR designs, Scott [11,12] has proposed that these cognitive constructs emerge from a general framework made up of what we will classify as necessary and sufficient conditions. The necessary conditions, discussed below, include cognitive, discursive, and material sources. However, it is only while meeting these three conditions that *ex post facto* co-operative activities can demonstrate that sufficient conditions have been met and thereby contribute to the emergence of sustainable cooperative cross-border governance networks in a borderland area. In Scott’s words “*…it is therefore necessary to differentiate between intentional policies, self-promotional rhetoric and actual co-operation experiences. It is, indeed, the latter that provides more sensitive criteria for judging co-operative efforts rather than a priori defined regional development concepts or planning initiatives.*”

A pervasive issue in the literature concerns the extent to which CBRs experience a unique identity. Scott’s typology identifies three “*sources of cross-border regionalism in Europe and North America*”. He continues that these are ([12], p. 614):

**Cognitive** “*(p)rocesses of creating regional self-awareness: identification with common problems and development contexts as pre-condition for establishing communities of interest.*”

**Discursive** “*(t)he creation of ideological platforms and paradigms that provide political legitimacy and orientation to cross-border regionalism.*”

**Material** “*(i)nstitutional frameworks: resources and incentives that encourage cross-border cooperation.*”

Thus, it is important to note the differentiation between these *a priori sources* of CBR regionalism and ex post facto *actions* that demonstrate their *true* existence.

This distinction between “necessary” and “sufficient” conditions is also contained, albeit with slightly different purpose, in Ali. He coins the term “*consensus catalysis*” to describe the phenomenon of cooperation that occurs on environmental matters when affected national parties perceive, and act upon, potential mutual gains. He employs the chemical analogy of “catalysis” to describe the transition from necessary to sufficient conditions ([8], p. 166):

*Just as chemical catalytic processes require specific temperature or pressure criteria for efficacy, an active negotiation climate with appropriate mediation may be required to make the parties realize the mutual gains from keeping environmental matters in mind*.

In Ali’s analogy, temperature and pressure are representative of a priori or necessary conditions. Catalysis, in an environmental management milieu, and according to game theory, can occur as a result of “aversion” to the outcome of non-cooperation *i.e.*, environmental degradation. Thus the bridge, between Scott and Ali, lies in recognizing that sustainable cross-border governance requires a transformative event, or set of actions.

### 2.3. Catalyzing Sustainable Consensus

Utilizing issues of cross-border environmental resource management based on dilemmas of common aversion represents the key element in Ali’s goal of catalyzing a sustainable consensus, thus moving from a position of non-cooperation or conflict to one of growing and sustainable cooperation. Basically, this hypothesizes that using a game theoretical approach two non-cooperating nations can begin the process of conflict resolution through cooperation on resource management issues given the aversion of each side to the alternative outcomes. In a sense this represents a “confidence builder” that can demonstrate the value of cooperation, even if unequal and/or unreciprocated from which neither party would never choose to move back to the earlier position of complete non-cooperation. This hypothesis is the result of an underlying logic: first, it is assumed that we are dealing with conflicts with multiple causality and that there is no absolute resource scarcity or zero sum game occurring; second, this leads to a reinterpretation of how resource management actually occurs between competing parties; third, the fact that resource management issues have become recognized as so important that there is a real aversion to ignoring them and a need to address them; and finally a leap of faith that success in the environmental arena will spill over into other areas creating a catalyzing process that results in broader cooperation and consensus even if unequal. Although Ali has focused on the national level, the same process can easily be applied at the regional. Thus, by using the metaphor of a catalyzing process, a successful outcome requires first meeting necessary conditions that create the potential to speed the outcome of a broader level of cooperation thus resulting in having met sufficient conditions for cross-border agreement.

If the only conflict between two places is over a single resource and/or this is an absolute resource where any change results in one party’s loss for the other’s gain (zero sum), then two things are clear. In the case of a single resource, there are no further conflicts to be addressed; as a result, talking of a catalyst for greater cooperation is moot. In the second case, if there is a zero sum game, then there is no incentive for change. Thus, we are dealing with “*…environmental issues as integral components of a multiple-causality conflict…(with) opportunities for using them to foster and sustain cooperation*” ([8], p. 166) across a broad spectrum of factors resulting in mutual gains for both players.

Garret Hardin [19] dealt with such absolute resource management referred to as a dilemma of common competitive interests. Based on Ali this situation can be illustrated with the following Table 1:

**Table 1:** Dilemmas of common competitive interests.

	Country A (Cooperation)	Country B (Non-Cooperation)
Country A (cooperation)	3,3	1,4
Country B (non-cooperation)	4,1	**2,2**

Values represent levels of preference for each country with 4 as greatest preference and 1 as least. The lower right corner is the only equilibrium point.

However, Ali concludes that this is an inaccurate portrayal of the situation. Given the level of environmental knowledge in this day and age, Ali states that “*…with increasing awareness of environmental issues, the dilemma is indeed one of common aversions. The common aversion…(being) the depletion of scarce resources.*” ([8], p. 167). This can be illustrated in Table 2:

**Table 2:** Dilemmas of common aversion and divergent interests.

	Country A (Cooperation)	Country B (Non-Cooperation)
Country A (cooperation)	3,3	**2,4**
Country B (non-cooperation)	**4,2**	2,2

Values represent levels of preference for each country with 4 as greatest preference and 1 as least. The lower left and upper right corner are both equilibrium points.

Without going into great detail on game theory itself in such a brief paper, it can be stated that under common competitive interests, the only equilibrium point in Table 1 is the southeast corner. This is a point from which neither party is compelled to move since all other outcomes are unstable because the opposing player could always decide not to cooperate and improve their own position while harming their opponent’s position. On the other hand, Table 2, one of common aversion and divergent interests, creates stable equilibrium outcomes in either the northeast or southwest quadrants. Although this rewards one party at a higher rate than the other, once chosen it is clearly preferable to total non-cooperation by both parties. Basically this demonstrates the clear advantage of some level of cooperation. The Koreas represent an example of how this might work in practice, where the South cooperates and receives a lesser benefit then the North, but feels compelled to do so since the alternative could be extremely bleak. Ali recognizes the unequal outcome of Table 2 but suggests that if time is factored into the game, then on some occasions party A could benefit more than B, and vice versa. Again thinking of the two Koreas one could argue that in fact South Korea is willing to forego immediate benefits for later rewards, but even if these other rewards do not come to fruition, it is still better off than complete non-cooperation. Another way of viewing this might be with multiple players, issues, and outcomes as well as across time, where unequal benefits are available to both sides to such an extent as to temper the results in any one instance. However, the one thing that is clear is that in Table 2 cooperation by any one party will definitely benefit both parties.

The third assumption is the relative importance of resource management issues *vis-à-vis* other contentious issues and therefore the desire to address these issues regardless of other outstanding ones. Certainly with the most basic resources such as clean water, air, and land, one is left with little to dispute. However, what this leaves unanswered is the relative importance of resources in a given geographic context. Certainly water will be a more pressing issue in an arid region then one with an overabundance of precipitation. Likewise proximity to impacts is also relative. For example, island nations have much more to fear from global warming and sea level rise then large continental ones. Thus this remains a less theoretically certain assumption, and an issue we will return to in our final discussion.

The final assumption, that success in the environmental arena will spill over into other areas of conflict and create a catalyzing sustainable consensus is both the most important and most contentious of the lot. Ali provides a number of arguments as to why this will be the case. The first argument rallies around science and fact. If resource issues are accepted to be real threats of which all parties are adverse to and unable to unitarily address, this can help to depoliticize the discussions and move them onto a more objective footing. Important in this process would be to capitalize upon the mutual aversion all sides have for the threat. However this also raises the issue of time. Whatever agreement is hammered out must include time, thus addressing not only how the benefits will be realized today but also how future benefits will be shared; this is where sustainability enters the mix. A second way of understanding the importance of time is the view of the future by both parties. Ali cites Axelrod ([20], p. 126) as noting that “*mutual cooperation can be stable if the future is sufficiently important relative to the present*”, what Axelrod calls the “*shadow of the future*”. This is assumed to be true since the impacts on the environment of current human activity tend to be felt more in the future rather than the present. Ali then concludes that:

*If potential adversaries are able to think of future consequences of present actions because of their common aversion to environmental harms, there is a greater likelihood that they may also bring the same outlook to other points of contention*.

([8], p. 168)

In summary by dealing with resource management issues through the lens of common aversion, parties are able to create agreements leading to sustainable activities that not only provide for a better environment both present and future but also lay the groundwork for further agreements in other areas.

## 3. The Setting: The Fraser Lowland

Great commonality exists in the Fraser Lowland environmentally, culturally, and historically and has for millennia despite its recent division between two nation states. As a result this has all the hallmarks for the development of a CBR. In a previous paper utilizing a cross border Delphi study, the authors have demonstrated that in fact all of Scott’s necessary conditions have been met [13]. However, this leaves open the issue of meeting the sufficient conditions. It thus becomes the task of this study, using the lens of Ali’s hypothesis of cooperation in resource management as the catalyst for sustainable consensus, to evaluate whether or not concrete activities and evidence can be identified to demonstrate that sufficient conditions have also been met.

In this exploration we limit the scope of our study to activities surrounding a single event and the spatial area most impacted by it; a proposed electrical generation plant, Sumas Energy 2 (SE2), adjacent to the American side of the border at Sumas, WA. The spatial extent of the study is the major impact area based on a combination of physical characteristics of the air shed and location of political decision makers which roughly forms an ellipse whose major axis runs from Bellingham, WA to Abbotsford, BC (refer back to Map I). The value of focusing on this particular event is that it is small and local enough to be carefully dissected for evidence of the kind of synapsis that Leresche and Saez define as crucial to an active and sustainable CBR and yet to not be of great interest to larger but more distant actors such as provincial, state, or national capitals, or even Vancouver, BC. If such confidence building activity occurs at this level, then one might look further afield to see if the catalytic process is spreading throughout the region.

### 3.1. Geographic Context

The Fraser Lowland is of relatively flat terrain, measuring approximately 3500 kilometers [21]. It is delimited by mountains to the north, south and east, and the Strait of Georgia to the west creating a confined air shed divided nearly equally between the US and Canada.

This region is dominated by Metro Vancouver’s population of 2.1 million. However, on the Canadian side, this study focuses on the more rural Fraser Valley Regional District (FVRD) to the east, encompassing the communities of Abbotsford, Chilliwack and Mission with a combined population of approximately 260,000. The 2.4 million people north of the border, dwarf the approximately 190,000 south. The American portion of the region is entirely contained within northwestern Whatcom County, dominated by the city of Bellingham. Despite the population imbalance, the Fraser Lowland as a whole is characterized by high rates of population growth and attendant economic activity. Whatcom County’s 2000–2006 growth rate was 11.5 percent [22] and compared to a five year 8.2 percent increase in the FVRD [23].

At the macro scale, the Fraser Lowland lies at the geographic epicenter of two larger, overlapping, cross-border regions: Cascadia and the Georgia Basin/Puget Sound (or, more narrowly, Vancouver-Seattle) region. Academic discourse on Cascadia has attempted to deconstruct the meaning of this indistinct but emotionally charged space [18,24,25,26,27]. In contrast, the Georgia Basin/Puget Sound is usually the focus for studies on the development of joint cross-border governance infrastructure, often in comparative analysis with other CBRs [10,28,29,30,31]. Regardless of how it is defined, literature on the region ignores activity in close proximity to the border. Moreover, the tendency is to focus on evidence of cross-border institutions, to support an argument about the material significance of a CBR.

Our micro scale analysis of activity that essentially occurs immediately adjacent to the border therefore stands in contrast. For those living on the border, the line is a negotiated entity that forms an implicit component of place and space [32]. When the boundaries of a CBR coincide with a local bioregion the border plays a role in the environmental management drama that inevitably ensues. Such was the case in the early years of this decade in our study area. A proposal, to build SE2, a second, gas-fired, electrical generation plant in Sumas WA, approximately eight kilometers from Abbotsford BC City Hall, generated a protracted legal battle and grassroots furor over diminished air quality, which lasted from 2000 until 2006 [33]. Although the protest had bi-national elements, it was heavily weighted on the Canadian side of the border. In the absence of an international air quality treaty in this part of North America, or any institutionalized dispute resolution mechanism, the proposal bounced back and forth across the border before it finally came to rest, just shy of the Canadian Supreme Court where anti-SE2 interests prevailed. The power plant proposal was eventually withdrawn. However, it is as much this dispute that triggered this micro scale study as its outcome.

### 3.2. Overview of the SE2 Decision Problem

In the late 1990s, National Energy Systems Co. (NESCO) of Kirkland, Washington (WA), began planning SE2, a 660 megawatt natural-gas powered electric generation plant on a 37-acre industrially-zoned lot in the small border town of Sumas, Washington (population of 1265) a half-mile from the Canadian border and Abbotsford, BC. The proposed plant would include an associated 200+ kilovolt transmission line, and as a merchant plant could potentially supply any market demand along the west coast international grid even as far south as Mexico. While the SE2 plant would have represented one of the cleanest generation plants in the region, plant operation still would have exacerbated local environmental problems, primarily the air shed. Further, most of these impacts would have been felt locally in the Fraser Lowlands and especially in the Abbotsford and Sumas region.

A timeline of major events, decisions, and players in the struggle that ensued, whether their focus was on energy or environmental security, and on which side of the border decision making power resided are outlined in Table 3. A brief discussion of these events follows.

**Table 3:** Power plant events timeline, security focus, and decision location.

Year	Event	Security Focus	Decision Location
1990	NESCO receives permits from City of Sumas, WA to construct SE1 a small 120 mw co-generation power plant	Energy	US
1998	SE1 effluent flows north across border to Abbotsford BC treatment facility	Environment	US & Canada
1999	NESCO files initial SE2 plans to EFSEC requesting permits for a large dual natural gas and diesel fueled 660 mw power plant.	Energy	US
2000	Grassroots campaign in opposition to SE2 in US and Canada	Environment	US
2001	EFSEC unanimously rejects initial plan in 0–11 vote	Environment	US
2001	NESCO submits a revised SE2 plan for a natural gas only power plant	Energy	US
2002	EFSEC approves SE2 permits by unanimous vote of 12-0	Energy	US
	BC and Environment Canada challenge US-EPA permits for SE2	Environment	US
	BC requested NEB to expand its normal jurisdiction to also consider environmental effects in the SE2 review process	Environment	Canada
	NEB declares that the review for connection of SE2 to the power grid can also include the environmental effects of the plant itself	Energy & Environment	Canada
2003	US-EPA rejects BC and Environment Canada’s challenge	Environment	US
	NEB rules that connection for SE2 to the power grid is not environmentally damaging	Environment	Canada
2004	NEB rejects request for SE2 permit to connect to Canadian power grid citing local Canadian environmental impacts of US plant itself	Environment	Canada
	NESCO appeals NEB decision to Canadian Federal Court of Appeal	Energy	Canada
2005	Canadian Federal Court of Appeal unanimously upholds NEB decision.	Environment	Canada
2006	NESCO decides not to appeal the Court decision to the Supreme Court of Canada withdraws application for SE2	Energy	US

The City of Sumas supported SE2 in an attempt to enhance long-term economic development and tax base expansion by leveraging its geographic proximity to Canada and expand on the success for the much smaller SE1 co-generation plant (only 120 megawatts) built in 1991. Given the city’s relative isolation from American transportation and power networks (over 20 miles from the nearest Interstate) a link with the Canadian economy appeared promising for a number of reasons. These included proximity to the Canadian branch of the North American power grid (only five miles to the north) and access to a Canadian natural gas trunk line. Hence, NESCO filed an application with the State of Washington’s Energy Facility Site Evaluation Council (EFSEC) to build the $400 million plant in January 2000.

Prior to all this, the City of Sumas had negotiated a unique 20 year cross-border arrangement with the City of Abbotsford to process sewage that included effluent from SE1. The sewage began to flow north in 1998. In the latter 1990’s, NESCO sought to expand the sewage deal, in preparation for the infrastructural requirements of SE2. The City of Abbotsford provided an agreement in principle in 1998, “*to approve Abbotsford’s James sewage treatment (facility) receiving the waste water from SE2, subject to receiving more information about the project*.” [34]. When that information appeared, in early 2000, Canadian public opinion quickly galvanized against the plant, as did the Abbotsford city council.

Fraser Valley health, school, and community groups were especially alarmed by the potential and current impact of emissions on human health and on the already stressed and fragile air shed around Abbotsford. A variety of factors already contributed to exceeding air quality objectives in the Lower Fraser Valley such as ozone and inhalable particulates (PM10); these factors included transportation (automobile use) and industry emissions, intensive agriculture (which generates high ammonia levels), and population growth [35]. In addition, the region’s meteorology and topography also contributed to conditions which would exacerbate the impact of additional emissions. Thus, the region’s air shed was already stressed and residents were suffering a range of health effects [36]. In particular, it was noted that asthma rates for children in the Lower Fraser Valley of British Columbia were among the highest in Canada [37]. Citizen groups were concerned that an increase in emissions of sulfur dioxide, nitrous oxides, ammonia and other pollutants would intensify health hazards. Concerns were also raised about the burning of diesel oil by backup generators, and emissions of lead, mercury, arsenic, cadmium, dioxins, and sulfuric acid mist.

Despite these concerns, a number of scientific assessments projected that the emissions of the SE2 power plant would not significantly cause Canadian air quality objectives to be further exceeded [38].

Further, as noted by the Canadian Federal Environment Minister David Anderson in July 2000: “*Burning natural gas for power is better than burning coal. Sumas2 would create a fraction of emissions now produced by existing pollution from vehicles and sources such as (BC Hydro’s) Burrard Thermal*” ([39], p. 5).

After numerous hearings and public debate, on February 16, 2001, EFSEC, by a vote of 0 to 11, recommended that the governor deny SE2 in large part because the proposal involved backup diesel generators. In addition, EFSEC raised other concerns about the project including air quality impacts, greenhouse gas emissions, water quality and quantity impacts at local wells, and the risk of increased flood hazard. In short, EFSEC [40] argued that “*environmental costs outweigh the energy benefits*”. Rather than risk final rejection by the governor [41], the original proposal was withdrawn by NESCO. In June 2001, a revised design was submitted, which removed the diesel backup provision. This plan was eventually approved by EFSEC, on a vote of 12 to 0 in May of 2002, and subsequently endorsed by Governor Gary Locke on August 23, 2002.

After Governor Locke’s approval of the project, Canadian government and opposition groups, supported by their American allies, appealed to higher government bodies, including the US EPA, to deny the final Environmental Impact Statement on obscure technical grounds. This was rather quickly and soundly rejected. Second, citizenry of BC turned to the National Energy Board of Canada (NEB). The role of the NEB was to issue the permit for connecting SE2 onto the power grid in Abbotsford. Traditionally the NEB limited its review to the direct impacts of power lines themselves. However, in the case of SE2, at the urging of BC, the NEB agreed in late 2002 to look at both the impact of the power line and the power plant to supply it, even though the power plant would be located in the US. This critical decision meant that a Canadian federal institution was effectively setting itself up to dictate environmental if not economic policy to their southern neighbor: without a connection to the Abbotsford power grid, the economic viability of SE2 was questionable. To a certain extent this moved the Canadian regulatory border south of the international border. After announcing in December of 2003 that the power line itself had an acceptable impact, in March 2004 the NEB rejected NESCO’s application on the grounds that the power plant itself would have adverse impacts on the local region in Canada.

### 3.3. Competing Interests or Common Aversion

To initiate the resource management lead catalyst for sustainable cross-border consensus a number of conditions must be met: first, it must be clear that there is multiple causality and no absolute resource scarcity. Despite the health concerns expressed on both sides of the border regarding current or potential levels of pollution, one of the greatest single contributors especially so in more heavily and densely populated Abbotsford—automobile usage—hardly seemed to be on anyone’s radar screen. Rapid population growth and automobile use has been booming on both sides of the border. Hence, one must conclude that despite the impacts of this growth the region has yet to reach the level of a zero sum game and given the large number of air pollution sources it certainly faces multiple causality.

Ali’s next two points overlap somewhat in the SE2 controversy. There is an aversion to air pollution, but it seems to be without a broad or long term focus. It is clear that many on both sides of the border opposed SE2 but there were not equal calls to control the growth of other sources of air pollution. It appears that cross-border air management has remained more a dilemma of common competitive interests. The goal is still growth and all are only starting to recognize the finitude of the air shed, but the plan for the immediate future seems to be to protect as much as possible for one’s own use and deny your neighbor the right to it. This hardly seems like a desire to address resource management policy in regards to the air shed first and foremost and does seem to be heavily focused on the present, not the future.

## 4. Discussion

To date we conclude that there is little to no evidence from the events surrounding SE2 to suggest a process of catalyzing sustainable consensus has occurred around this resource management issue. A quick overview of Table 3 alone indicates that the major American policy focus was on energy and Canadian focus on the local environment. Despite this it is true that there is some evidence of cross-border understanding such as the initial EFSEC unanimous rejection of the first SE2 proposal with diesel back-up generators. However on the whole one would have to typify this as a conflict where “the last man standing” became the victor. In fact even though NESCO eventually withdrew they could have continued the fight, by filing an appeal to the Canadian Supreme Court or even to the US Federal Government, suggesting that NAFTA was being thwarted. The likely outcome of such a gambit is not clear. What is clear is that cooperation was not key to the outcome on either side of the border. More likely exhaustion and changing economic conditions including higher natural gas prices making SE2 less profitable caused NESCO to withdraw from this conflict.

As suggested in the previous section, this still looks like a dilemma of common competitive interests rather than one of common aversion of divergent interests. It smacks more of NIMBYism then of a desire to address cross-border resource management. It is clear that many stakeholders do view the air shed as a finite and stressed resource and already are concerned about current impacts, but on neither side of the border has a critical mass been met that has caused a complete evaluation of the situation. Instead band-aid solutions are being suggested and the “border as a shield” has been manipulated rather handily in this particular instance, to be discussed further, below.

As a result, a main conclusion of this study is to suggest that even if Ali’s hypothesis is correct it is premature to test in this part of the Fraser Lowland concerning the stressed air shed. However, perhaps another way of interpreting the outcome is to say that there may be a different resource that is operating in the aversion rather than competitive arena. Water might be another resource to investigate in the same micro area since Abbotsford and Sumas sit squarely on top of a shared cross-border aquifer and surface drainage, and the former flows north to south across the border and the latter in the opposite direction. Although it is not the purpose of this paper to fully investigate such an issue it should be mentioned, as noted earlier, the two towns share a single waste water treatment plant. Since the natural drainage between the two towns flows south to north, Abbotsford sits down stream of Sumas’ effluent. A decade ago an agreement was signed whereby Sumas contracted to use Abbotsford’s treatment plant as a way to address both local and cross border sewage issues. However, this clear case of cooperation may be unraveling. As part of the fallout of SE2, Abbotsford has considered terminating the contract. This is seen as a way to prevent a replay of SE2 by denying an essential ingredient to industrial expansion in Sumas. If this comes to pass it places in doubt a critical level of aversion in regards to this resource as well.

Although Ali stresses resource management as the key to creating the catalytic reaction, why does this need to be the case? Is there some reason that aversion to choices that cause economic damage could be any less compelling? Much has been made of attempts to speed the flow of goods back and forth across the border and unique forms of cooperation have occurred here, including the construction of a truck staging area on the Canadian side of the Sumas-Abbotsford border crossing using US Federal highway dollars [42]. This certainly suggests an additional avenue to be explored.

Finally, let us return to the border as a shield and how it has complicated US-Canada relations over the last century and a half. A careful read of the SE2 conflict suggests that the border as a shield was rather deftly employed during this series of events. Thus, rather than directly engaging Sumas and its concerns and attempting to meet these needs in a manner more favorably disposed to the air shed, Abbotsford appealed to a number of actors in Canada to help them enforce their view of situation, specifically NEB and the Canadian courts. In addition, one must realize that many scholars [32,43] have seen the shield metaphor as central to Canadian identity (“*being Canadian means not being American*”). Might this tend to somewhat temper or even eventually thwart the development of a sustainable catalyzing process leading to consensus between the two countries? These are issues important to raise, but beyond the scope of this paper to fully answer.

## 5. Conclusions

In our micro level study in the Fraser Lowland, competing security interests remain: environmental and energy based. Although Canadian environmental policy appears to triumph and at least in part create trans-border consequences; the American players’ acquiescence appears to be more a result of changing energy and commodity markets then agreement. Basically the dispute remains unresolved. As a way out of this corundum we turned to Ali and cross-border theory.

Ali provides a tantalizing hypothesis, one which provides a possible way of leading to an active and sustainable CBR. However, we fail to find evidence of movement towards a solution based on common aversion and catalyzing behavior. Perhaps, as noted previously this is simply premature. Perhaps the stress on the air shed is not yet great enough to move this to a decision based on aversion. Or maybe another resource will provide the venue, or perhaps we should move beyond strictly environmental resources and consider economic or other activities as well. One thing that we do not suggest is to throw the baby out with the bathwater. Although we are unable to conclude that sustainable conditions for an active and sustainable CBR governance have been met in the Fraser Lowland, in the author’s opinions this does not mean that we should give up on the idea of creating a catalyzing sustainable consensus through an aversion based cross-border natural resource policy. Our recommendation is to continue to look. Ali’s ideas provide excellent food for thought and certainly more clearly address the missing link in CBR development: the movement from necessary to sufficient conditions. However, this stated one should also be cautious about ascribing universality to Ali’s hypothesis. Perhaps the process is a bit more complex and dependent on local issues, history, and tradition than at first suggested. Might the European Union provide a richer source of CBRs to view through Ali’s lens? Maybe there are additional necessary conditions to be met before we can move on to the sufficient conditions, perhaps the border can no longer be viewed as a shield meaning that Scott’s local cross-border cognitive condition has to be developed to the extent to overcome previous practices of hiding behind or needing to hide behind the border as has been so common in the American-Canadian context.
